# EIT Ambivium, Linea Semilunaris, and Fulcrum Abdominalis

**DOI:** 10.3389/jaws.2023.12217

**Published:** 2023-12-22

**Authors:** Maaike Vierstraete, Jose Antonio Pereira Rodriguez, Yohann Renard, Filip Muysoms

**Affiliations:** ^1^ AZ Maria Middelares, Ghent, Belgium; ^2^ Hospital Universitario del Mar, Barcelona, Spain; ^3^ General and Digestive Surgery, Université de Reims Champagne-Ardenne, Reims, France

**Keywords:** anatomy, Linea Semilunaris, rectus sheath, component separation, abdominal wall anatomy

## Abstract

Building upon the recent advancements in posterior component separation techniques for complex abdominal wall hernia repair, highlights the critical importance of a thorough understanding of the abdominal wall anatomy. To address anatomical concepts with a pivotal role in hernia repair, we propose two new terminologies: “EIT Ambivium” referring to the lateral border of the rectus sheath, and “Fulcrum Abdominalis” demarcating the point where the Linea Arcuata intersects with the EIT Ambivium.

## Introduction

The recent advancements in complex abdominal wall repair underscore the critical significance of a comprehensive understanding of the abdominal wall anatomy. At the core of this comprehension is the recognition of the pivotal role played by the lateral border of the rectus sheath, formed by the convergence of the three lateral abdominal wall muscles. It has become increasingly common for surgeons to refer to this structure as the Linea Semilunaris, however this is incorrect. Our goal was to elaborate on the correct anatomical terminology while introducing two new anatomical concepts: EIT Ambivium and Fulcrum Abdominalis.

## Anatomy Abdominal Wall

**FIGURE 1:**
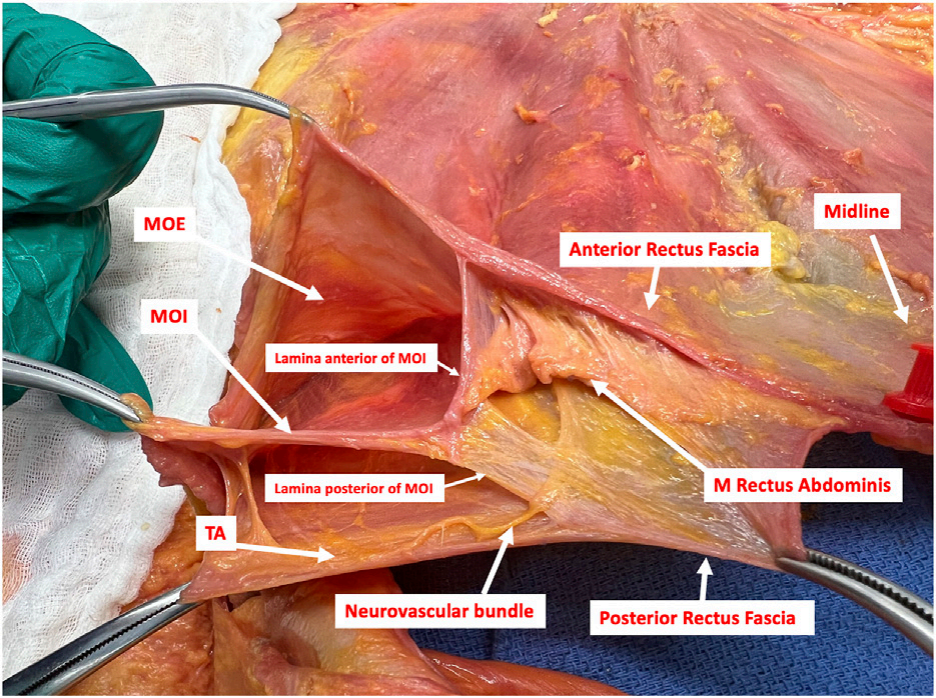
Transverse dissection of the right side of the anterior abdominal wall in the epigastric region showing the EIT Ambivium of the Musculus Obliquus Externus (MOE), the Musculus Obliquus Internus (MOI) and the Musculus Transversus Abdominis (TA). The lateral border of the rectus sheath is formed by the splitting of the MOI into its anterior and posterior lamina. Those are fusing with the aponeurosis of the MOE anteriorly and the musculoaponeurotic TA posteriorly to form the anterior and posterior rectus sheath respectively. A neurovascular bundle, with a terminal branch of an intercostal nerve for innervation of the rectus muscle, is seen between the MOI and the TA. This bundle penetrates the posterior lamina of the MOI in a medial direction towards the rectus muscle (Picture from a cadaveric dissection conducted at the Anatomy lab of Professor Yohann Renard at the University of Reims, Champagne-Ardenne, France).

The anterior abdominal wall consists of a medial compartment and a lateral compartment. Within the medial compartment, the rectus muscle has its origin at the pubic bone in the pelvis and extends to insert onto the costal cartilages of ribs V–VII, as well as the xiphoid process. The rectus muscle is surrounded by the rectus sheath, comprising an anterior and posterior layer formed by the aponeuroses of the lateral abdominal wall muscles. The anterior and posterior rectus fascia unite medially of the rectus muscle creating the linea alba along the midline. This relationship is depicted in Figure 1.

**FIGURE 2:**
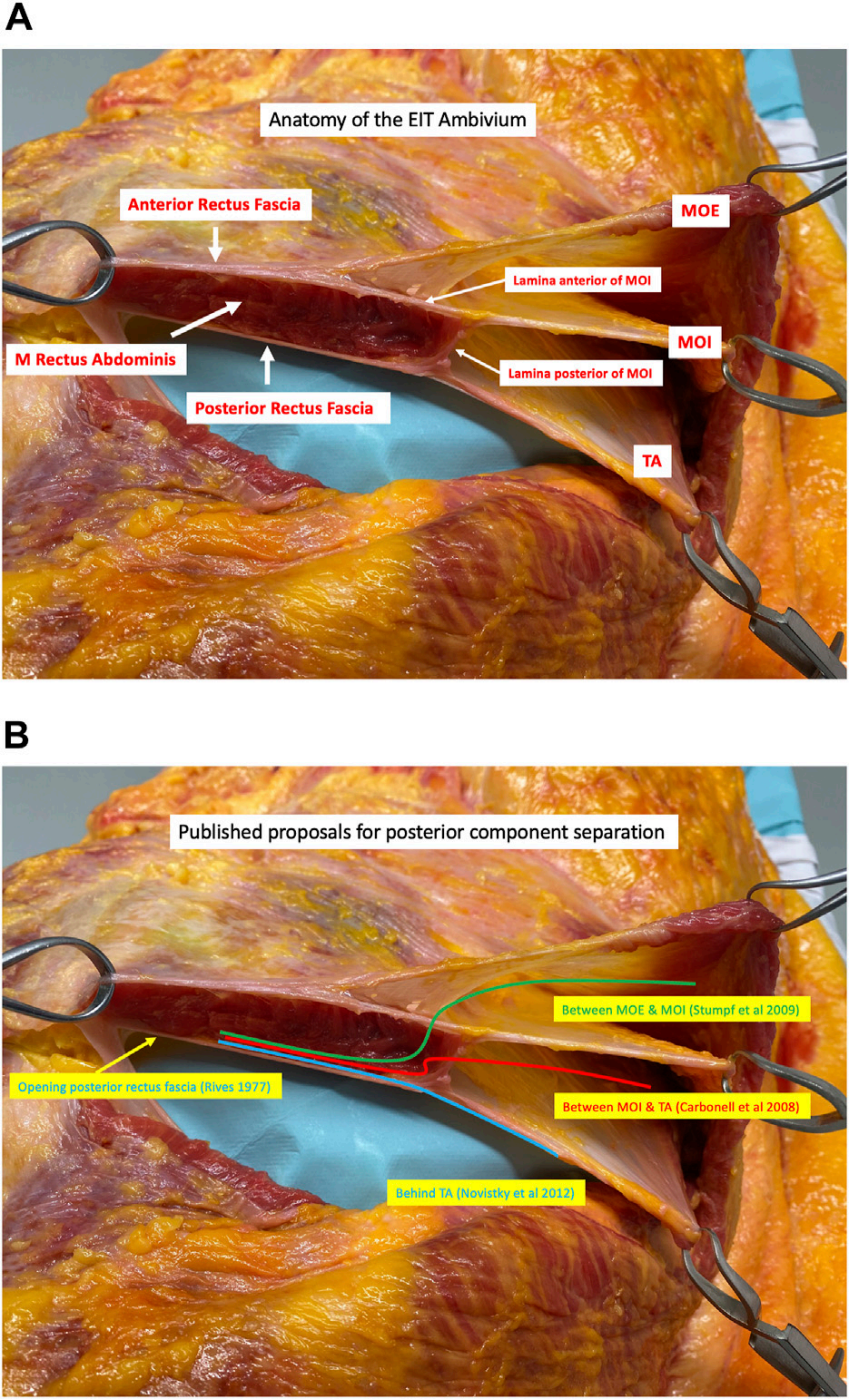
**(A)** Transverse dissection of the right side (viewed from a cranial perspective) of the anterior abdominal wall in the epigastric region showing the junction of the Musculus Obliquus Externus (MOE), the Musculus Obliquus Internus (MOI), and the Musculus Transversus Abdominis (TA). The lateral border of the rectus sheath is formed by the division of the MOI into an anterior and posterior lamina. These laminae then fuse with the aponeurosis of the MOE anteriorly and the musculoaponeurotic fibres of the TA posteriorly to form the anterior and posterior rectus sheath respectively. The acronym EIT Ambivium is proposed: MOE–MOI–TA, to designate the lateral border of the rectus sheath (Picture from a cadaveric dissection performed at the Anatomy lab of Professor Yohann Renard at the University of Reims, Champagne-Ardenne, France). **(B)** Each posterior component separation technique starts with a medial incision of the posterior rectus fascia next to the midline allowing for a dissection in the retrorectus plane, referred to as Rives-Stoppa dissection (1). The most superficial posterior component separation technique is performed by incising the lamina anterior of the MOI to enter the inter-oblique plane (2). A layer deeper, the lamina posterior of the MOI can be incised, accessing the plane between the MOI and the TA (3). The deepest posterior component separation technique, known as Transversus Abdominis Release (TAR), involves making an incision in the lamina posterior of the MOI approximately 1 cm medial to the course of the neurovascular bundles and about 1.5 cm medial to the junction. Subsequently, the contribution of the TA muscle to the posterior rectus fascia is incised, which is muscular in the upper part of the abdomen and aponeurotic in the lower part (4).

Within the lateral compartment, the abdominal wall comprises three distinct muscles, each characterized by a unique orientation of their muscle fibres: the Musculus Obliquus Externus (MOE), the Musculus Obliquus Internus (MOI) and the Musculus Transversus Abdominis (TA). Understanding the interrelationship between the lateral muscles and the medial compartment is of paramount importance for surgeons to understand the surgical anatomy. This comprehension is essential to adequately perform component separation techniques during complex abdominal wall repairs. Figure 2A demonstrates that the lateral border of the rectus sheath is not a simple line, but rather a junction where all three lateral abdominal muscles come together. In the upper abdomen, the anterior rectus fascia is formed by the fusion of the anterior lamina of the MOI with the aponeurosis of the MOE, while the posterior rectus fascia results from the fusion of the posterior lamina of the MOI with the musculoaponeurotic TA. In the lower part of the abdomen, the contribution of the TA is aponeurotic and no longer muscular.

## Component Separation Techniques

Component separation techniques, commonly used to address large incisional hernias, can be broadly classified into anterior and posterior approaches, depending on which muscles of the lateral abdominal wall that are divided. Regarding posterior component separation techniques, each technique starts with a medial incision of the posterior rectus fascia next to the linea alba allowing for a dissection in the retrorectus plane towards the lateral border of the rectus sheath. This widely recognized dissection technique is commonly referred to as the Rives-Stoppa dissection [1]. In Figure 2B, different pathways proposed for posterior component separation techniques are illustrated.

In a landmark paper authored by Stumpf et al. from the research group led by Volker Schumpelick in Aachen, Germany, a potential posterior component separation technique to connect the medial and lateral compartment of the anterior abdominal wall is outlined [2]. This technique involves establishing a connection between the retrorectus plane and the lateral intermuscular plane situated between the MOE and MOI. This connection is achieved by surgically incising the lateral edge of the rectus sheath, accomplished by transecting the anterior lamina of the MOI. Interestingly, during a similar timeframe, Carbonell et al. published an alternative way to establish a connection between the retrorectus plane and the lateral compartment [3]. Their method involved making an incision in the posterior lamina of the MOI at the lateral border of the rectus sheath. By doing so, the intermuscular plane between the MOI and the TA muscle was reached.

Expanding upon the accumulated knowledge of surgical anatomy and the notion of posterior component separation techniques, a ground-breaking innovation in abdominal wall reconstruction was introduced through a seminal paper by Novitsky et al [4]. This technique preserves the integrity of the lateral border of the rectus sheath by selectively incising the posterior lamina of the MOI and the musculoaponeurotic TA muscle. Notably, this transection is carefully performed medial to the neurovascular bundles to ensure the preservation of the neurovascular bundles responsible for innervating the rectus muscle. The latter being the fundamental principle behind this posterior component separation technique, known as Transversus Abdominis Release (TAR). TAR enables the disconnection of the posterior rectus sheath from all the lateral muscles, facilitating the placement of a broad mesh behind both the retrorectus plane and the lateral retromuscular plane, specifically behind the TA muscle. Moreover, TAR allows for a substantial medialization of the anterior and posterior midline planes, which plays a vital role for performing an abdominal wall reconstruction in cases of wide incisional hernias, eliminating the need to bridge the defect [5, 6].

## The EIT Ambivium *Aka* the Lateral Border of the Rectus Sheath

The recent breakthrough in posterior component separation techniques for complex abdominal wall repair highlights the critical importance of a thorough understanding of the abdominal wall anatomy. Central to this understanding is the recognition of the pivotal role played by the lateral border of the rectus sheath. This border is formed by the convergence of the three lateral abdominal wall muscles situated on the lateral side of the rectus muscle. Unfortunately, it has become increasingly common for surgeons to refer to this structure as the “Linea Semilunaris” in presentations, publications, videos, and social media. This anatomical misinterpretation defies our surgical heritage and serves as the primary trigger for this manuscript. Also, the lateral border of the rectus sheath does not resemble a “half-moon”, which is the inspiration behind the term “Linea Semilunaris”. In our opinion, this misconception stems from the lack of a proper name for the lateral border of the rectus sheath as an anatomical landmark. Recently, during a meeting held at IRCAD in Strasbourg, Victor Radu proposed the term “the surgical Linea Semilunaris” as a more accurate descriptor. However, in our opinion, this term lacks sufficient distinctiveness. To address this, we propose a novel acronym to designate the lateral border of the rectus sheath: the EIT Ambivium, which stands for MOE–MOI–TA junction.

## The Linea Semilunaris

The Linea Semilunaris, originally described by Adriaan van den Spiegel, a renowned Flemish anatomist born in Brussels in 1578, carries historical significance. Serving as a Professor of Surgery and Anatomy at the Univeristy of Padua in Italy until his demise in 1625, Van Den Spiegel made significant contributions to the field through his remarkable anatomical work titled “*De humani corporis fabricia libri decem tabulis aere icisis exornati*.” This work was published posthumously in 1627 by his pupil Casseri, initially without drawings, but later supplemented with illustrations in 1646 by Daniel Rindfleisch (Bucretius) using Casseri’s drawings. Within his work, Adriaan van den Spiegel described the Linea Semilunaris as *the line forming and marking the transition from muscle to aponeurosis in the transversus abdominis muscle* of the abdomen (“*circa quam tendines obliquorum abdominis musculorum incipent*”) [7].

The Linea Semilunaris, also known as the Semilunar Line, plays a crucial role in delineating the Spigelian fascia. This fascia refers to the portion of the aponeurosis situated between the Linea Semilunaris and the lateral border of the rectus muscle. Whenever there is a congenital or acquired defect in this Spigelian fascia, it can lead to the protrusion of a peritoneal sac, an organ, or preperitoneal fat, a condition known as a *hernia Spigelii or Spigelian hernia*. In 1764, Klinkosch described this type of Spigelian hernia; however, it was not until 1877 that Mollière used this eponym for it.

As per Wikipedia’s 2023 definition, the Linea Semilunaris is defined as: “*The Semilunar Line, Linea Semilunaris or Spigelian line is a curved tendinous intersection found on either side of the rectus abdominis muscle*. *The Linea Semilunaris corresponds with the lateral border of the rectus abdominis muscle*” [8]. What led to this misunderstanding and when did it arise? The current commonly used definition, which states that the Linea Semilunaris represents the lateral border of the rectus sheath where the lateral muscles merge to form the rectus fascia, is incorrect. In reality, the anterior rectus fascia comprises the aponeurotic contributions from the MOE and the anterior lamina of the MOI. Conversely, the posterior rectus is formed by the posterior lamina of the MOI and the TA muscle. While the contribution from the MOI and the lower one-third of the TA muscle is aponeurotic in nature, the upper portion of the abdomen consists of a muscular component from the TA muscle.

Therefore, it is evident that a significant disparity exists between the original description and the current misunderstanding of the Linea Semilunaris. In fact, each lateral abdominal wall muscle exhibits a distinct intersection line between its muscular and aponeurotic part. None of these lines bears any resembles to the lateral border of the rectus sheath, and only one of these lines possesses a semilunar shape, specifically the one formed with the TA muscle. This perspective aligns with the views expressed by other authors, like Skandalakis (2006) who emphasized “*We remind the reader that the convex Linea Semilunaris* (*Semilunar Line of Spieghel*) *is produced by and marks the site of transition from the aponeurotic part to the muscular part of the transverse abdominal muscle* [9].”

A more precise description of the lateral border of the rectus muscle would depict it as the convergence point between the anterior and posterior rectus fascia when approached from a medial perspective, or as the separation of the MOI into its anterior and posterior laminae when observed from a lateral viewpoint. Also, if the definition labelling the Linea Semilunaris as the lateral border of the rectus sheath were accurate, it would negate the existence of the Spigelian fascia, and consequently eliminate the occurrence of Spigelian hernias.

**FIGURE 3:**
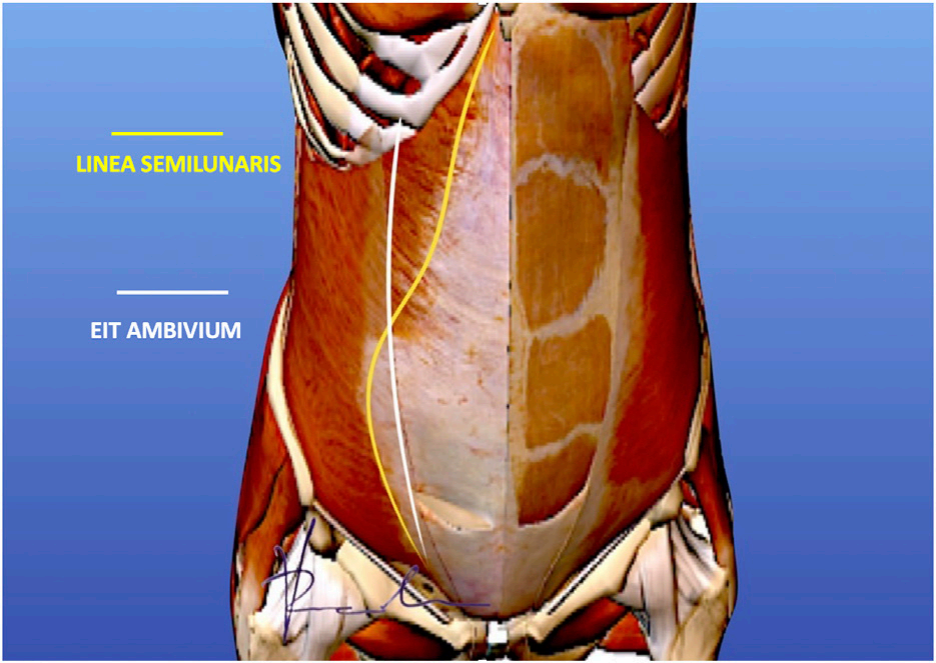
EIT Ambivium versus Linea Semilunaris. The white line representing the lateral border of the rectus sheath, should be distinguished from the yellow line which is the Linea Semilunaris as described by Adriaan van den Spiegel. Our proposal is to name the lateral border of the rectus sheath the EIT Ambivium to clearly distinguish this anatomical landmark from the Linea Semilunaris (Picture courtesy of Dr. Victor Radu).

**FIGURE 4:**
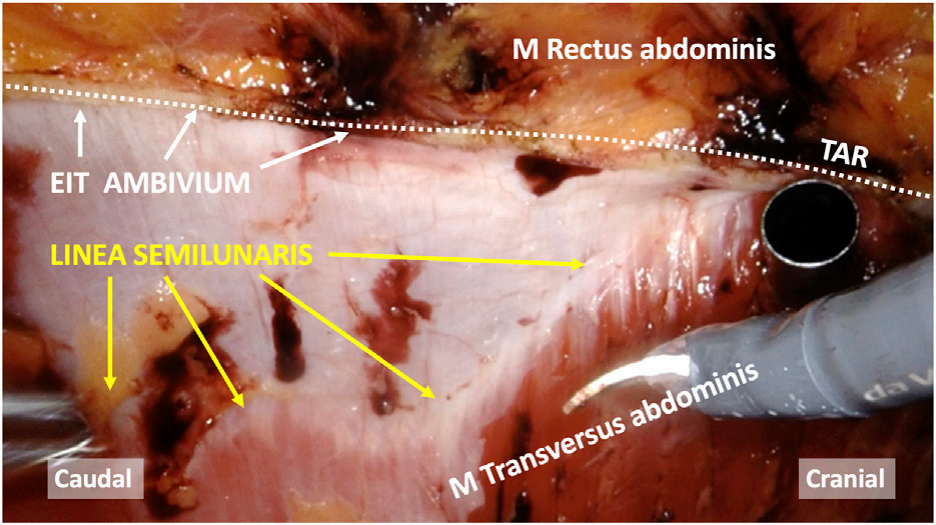
Intraoperative view of a robot-assisted Transversus Abdominis Release (TAR), highlighting the difference between the Linea Semilunaris versus the lateral border of the rectus sheath (EIT Ambivium). The Spigelian fascia refers to the aponeurosis situated between the Linea Semilunaris and EIT Ambivium.

We propose to use the acronym “EIT Ambivium” to refer to the lateral border of the rectus muscle, representing the convergence of the MOE, MOI, and TA. This designation enables a clear differentiation from the originally described Linea Semilunaris, which specifically denotes the line demarcating the transition between the muscular and aponeurotic part of the TA muscle. By introducing the EIT Ambivium terminology, we can precisely differentiate these two distinct anatomical landmarks (Figure 3). Additionally, the Spigelian fascia can be accurately identified as the fascia situated between the EIT Ambivium and the Linea Semilunaris at the level of the TA muscle (Figure 4). This naming facilitates a comprehensive understanding of the abdominal wall anatomy and provides a more detailed anatomical reference for abdominal wall surgeons.

## The Fulcrum Abdominalis

**FIGURE 5:**
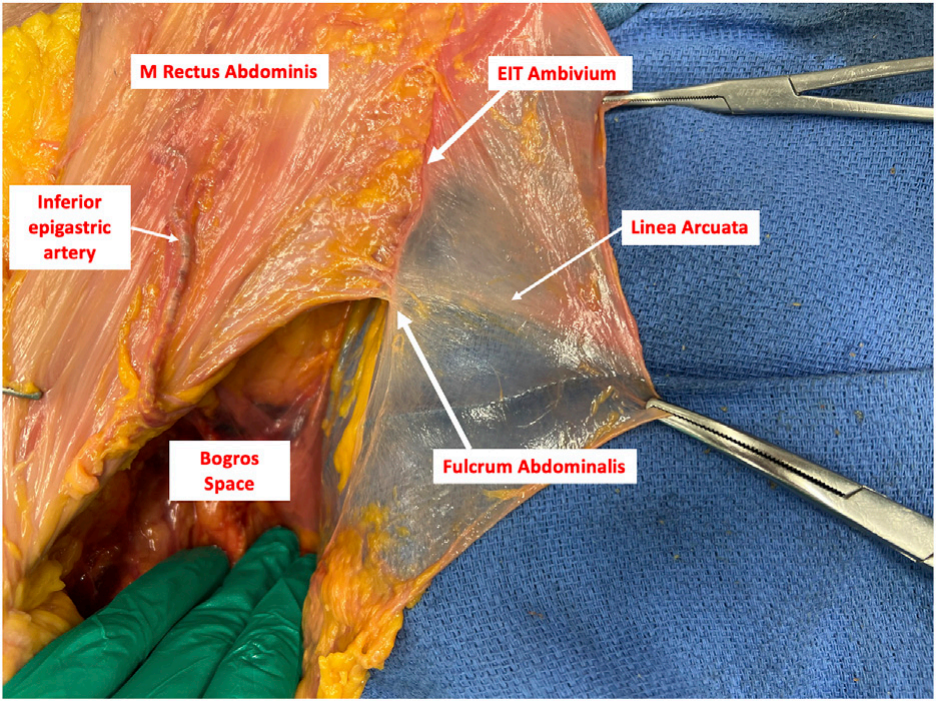
The Fulcrum Abdominalis serving as the intersection point between the Linea Arcuata and the EIT Ambivium.

An important anatomical feature in the anatomy of the abdominal wall is the termination of the posterior rectus sheath at the level of the Linea Arcuata. Below this line, the three lateral abdominal wall muscles fuse to form the anterior rectus fascia. The crossing point between the Linea Arcuata, and the EIT Ambivium, holds particular significance in innovative surgical techniques for abdominal wall reconstruction, such as TAR and enhanced totally extraperitoneal procedures (eTEP). Apparently, there is a consensus among several individuals regarding the significance of this particular anatomical landmark [4]. The same point is referred to as the lowermost point between the prongs of the recently described fatty trident by Garcia-Urena et al. [10]. To designate this critical anatomical point within the abdominal wall, we propose the term “Fulcrum Abdominalis” (Figure 5). By assigning a distinct name to this structure, we aim to enhance awareness about its existence, thereby recognizing its significance and facilitating its proper utilization in the future of abdominal wall surgery.

## Limitations

The limitation of our new terminology is that it was formulated by a limited group of surgeons. At the same time, the strength lies in the fact that the authors of this manuscript are advanced surgical anatomists which might enhance the adoption of the current proposal.

## Conclusion

The recent advancements in surgical techniques for complex abdominal wall hernia repair emphasize the importance of understanding the anatomy of the abdominal wall. To address the widespread misuse of the term “Linea Semilunaris”, we propose a new acronym “EIT Ambivium” referring to the lateral border of the rectus sheath. Additionally, we introduce the term “Fulcrum Abdominalis” as a novel anatomical landmark, signifying the point where the Linea Arcuata intersects with the EIT Ambivium. These two anatomical concepts, the EIT Ambivium and Fulcrum Abdominalis, play a pivotal role in driving innovative surgical approaches for complex abdominal wall reconstruction. Understanding these anatomical concepts is vital for achieving successful outcomes and promoting further advancements in abdominal wall surgery.
